# Household Income and Psychological Distress: Exploring Women’s Paid and Unpaid Work as Mediators

**DOI:** 10.3390/ijerph18126402

**Published:** 2021-06-13

**Authors:** Bonnie Janzen, Laurie-Ann Hellsten

**Affiliations:** 1 Department of Community Health & Epidemiology, University of Saskatchewan, Saskatoon, SK S7N 5E5, Canada; 2 Faculty of Education, The University of Winnipeg, Winnipeg, MB R3B 2E0, Canada; l.hellsten@uwinnipeg.ca

**Keywords:** mental health inequities, unpaid domestic labor, SES gradient, household division of labor, differential exposure, work stress, mediation

## Abstract

Research suggests that a socioeconomic gradient in employed adults’ mental health may be partially mediated by their work conditions. Largely ignored in this body of research is the potential role of unpaid domestic labor. The objectives of this paper were to determine whether socioeconomic disparities in mental health were present in a sample of employed, partnered mothers, and if so, identify the intervening mechanisms which contributed to the disparity. Participants for this cross-sectional study were 512 women recruited from an online research panel of residents living in Saskatchewan, Canada. Household income was the primary exposure and psychological distress was the dependent variable. Potential mediators included material deprivation, job control, job demands, work–family conflict, and the conditions of domestic labor. Descriptive analyses followed by simple and multiple mediation analyses were performed. Lower income was associated with greater distress, with material deprivation, work–family conflict, and inequity in responsibility for domestic work acting as mediators. These results suggest that in addition to more well-established mechanisms, the conditions of unpaid domestic labor, particularly how that labor is shared within households, may play a role in the genesis of mental health inequities among employed partnered mothers. Limitations of the study are discussed as are implications for future research.

## 1. Introduction

The personal, social, and economic costs associated with common mental health disorders and related symptomatology are considerable [1,2]. Many studies report a link between mental health and socioeconomic status (SES), with those of lower SES reporting a greater burden of mental health problems compared to their higher SES counterparts [3,4]. In research undertaken to explain this pattern, support has accumulated for the differential exposure hypothesis [5], which posits that lower SES individuals are more likely than those of higher SES to encounter adverse material, psychosocial and/or behavioral exposures, which in turn, increases their probability of psychological morbidity. Studies focused on employed populations have emphasised work as an important source of harmful psychosocial exposures, linking various characteristics such as low job control and effort–reward imbalance to both the development of mental health problems [6,7] and lower SES [8,9]. Considerable research suggests that the social gradient in workers’ mental health may be partially explained (i.e., mediated) by their work conditions [9,10], although results to the contrary have been reported. For example, studies have found that some work-related characteristics considered detrimental to mental health may be more prevalent among higher SES workers, such as psychological demands and work–life conflict [11,12,13], thus minimizing rather than contributing to SES inequalities in mental health [13,14].

Largely ignored in research attempting to elucidate work-related mechanisms involved in SES gradients in mental health is the role of unpaid domestic labor; that is, whether characteristics and qualities of housework and caregiving, just like paid work, may not only impact mental health, but also be shaped by socioeconomic circumstances [15,16]. Understudied, but not absent—a small body of research has reported more mental health problems among women involved in domestic labor that is demanding [17,18], routine [19], lacking in control [19], inequitably shared [20,21,22], high in demands and low in control [23,24], and imbalanced in effort versus reward [15]. Research further linking these stressful domestic work conditions to social inequalities in mental health is more limited. Although some earlier research explored unpaid household work as one potential pathway to SES disparities in women’s psychological distress [25], a key limitation was the use of crude indicators of domestic workload, such as household size and the presence of young children. Similarly, using Whitehall civil servant data, Griffin and colleagues [26] found lower levels of perceived control at home to be associated with both lower occupational grade and higher levels of depression, but the vagueness of the single-item question used to assess exposure created challenges in interpretation. In general, and very much in contrast to the extensive focus on measurement quality in the literature on paid work and health [27,28], research attempting to incorporate unpaid family work into the study of mental health inequities has been seriously limited by the use of unvalidated measures of key constructs.

There are some recent exceptions. Using a validated measure of effort–reward imbalance in unpaid family work [15,29], Sperlich and Geyer [30] reported less educated mothers perceived fewer rewards than their more educated counterparts, which partly explained the observed educational gradient in somatic symptoms; however, in contrast to rewards, more highly educated women reported greater domestic workload (i.e., effort) than those less educated. More recently, Janzen and Hellsten [16] described the development of the Family Work Quality Questionnaire (FWQQ), a multi-item measure assessing five psychosocial dimensions of unpaid domestic work: demands, autonomy, equity, social resources, and caregiving rewards. While all qualities were related to women’s mental health, only one subscale—equity—was also associated with educational attainment, with less educated women reporting less equity in responsibility for domestic labor compared to their more educated counterparts. The authors concluded that perceived inequity may be one potential mechanism linking lower education with greater psychological distress in a sample of employed partnered mothers; however, mediation was not explicitly tested.

This paper builds on Janzen and Hellsten [16], further examining social inequalities in psychological distress in the same sample of women by using household income as an indicator of SES and by including formal tests of mediation. The objectives of this paper were to determine whether an income gradient in psychological distress was present, and if so, identify the intervening mechanisms which contribute to the disparity. In addition to considering more conventionally included mediators of social inequalities in mental health (e.g., material deprivation, job control, job demands), the present study also examined the contribution of more novel exposures related to unpaid domestic work and the work–family interface.

## 2. Materials and Methods

### 2.1. Design and Participants

Participants of this cross-sectional study were recruited from an online research panel of approximately 10,000 residents living in Saskatchewan, Canada, a province of approximately 1 million people located in the Western region of the country [16]. Enrolled into the panel through a variety of print and social media sources, these members agreed to participate in online research on a continuing basis for nominal payment. In the spring of 2012, the survey was broadcast to a randomly selected sample of panel members who then answered several screening questions; respondents who indicated being 24–54 years of age, female, employed, and partnered with at least one non-adult child living in the household, were provided a link to the complete survey. We focused on: (1) 25–54-year-olds, as this is a period in the life course when women are most likely to be simultaneously engaged in childrearing and paid work roles [31]; (2) mothers, because childrearing is a core component of unpaid family work; and (3) partnered mothers, given previous research suggesting that the way couples share domestic labor seems to be important for women’s mental health [20,21,22]. During data collection, the socio-demographic profile of the participants was regularly examined to ensure that a broad socioeconomic spectrum of women was being sampled. Those who did not complete the survey by a pre-determined date were sent reminder emails with a link to the online survey. Approval was obtained from the university’s ethics review board prior to data collection.

### 2.2. Measures

#### 2.2.1. Dependent Variable

Psychological distress, the dependent variable, was assessed by the Kessler-6 (K6) [32], a 6-item measure requiring respondents to indicate on a 5-point Likert scale (1 = none of the time to 5 = all of the time) how frequently they experienced various symptoms of distress (e.g., hopelessness, nervousness, sadness) in the past month. Participants’ answers were summed, ranging from 6 to 30, with higher scores indicating greater psychological distress. Cronbach’s alpha for the scale was 0.88. Previous research provides evidence of the validity and reliability of K6 as a measure of nonspecific distress in community samples [32,33,34].

#### 2.2.2. Independent Variables

Primary Exposure

SES was measured using four categories of annual household income: ≤CAD59,999; CAD60,000–CAD89,999; CAD90,000–CAD119,999; and CAD120,000 or greater.

Mediators

Material deprivation was measured by asking participants to specify the degree to which they had experienced challenges in fulfilling basic financial-related needs in 11 different areas (e.g., housing, food, children’s clothing, transportation, etc.) [35]. Response categories ranged from not at all difficult (1) to very difficult (4). Scores were summed, with higher scores indicating greater material deprivation.

Karasek’s Job Content Questionnaire (JCQ) was used to assess two psychosocial dimensions of paid work, job demands, and decision latitude [28]. The job demands subscale (9 items) measures the pace, effort, and volume of work. Decision latitude (9 items) measures two dimensions: (1) decision authority (i.e., authority to make decisions concerning work); and (2) skill discretion (i.e., ability to use one’s skills in doing work). The questionnaire items were coded from 1 (strongly agree) to 4 (strongly disagree). All items were recoded in the same direction, and scores for each scale were calculated by summing the item scores. A higher score on each subscale indicated greater job demands and decision latitude. Cronbach’s alpha for decision latitude and job demands was 0.80 and 0.79, respectively. The validity and reliability of the JCQ has been extensively documented.

The psychosocial characteristics of unpaid domestic labor were measured by the Family Work Quality Questionnaire (FWQQ) [16], a self-report 28-item measure assessing five dimensions of unpaid family work: demands (i.e., time pressures and conflicting demands), autonomy (i.e., freedom over decisions), equity (i.e., perceived fairness in the division of family work), social resources (i.e., availability of people beyond the immediate family to provide assistance), and caregiving rewards (i.e., intrinsic gratification from caregiving responsibilities) (Table A1). A detailed summary of the FWQQ’s development and evidence of reliability and validity were provided in Janzen and Hellsten [16]. Briefly, participants were provided with a definition of family work and asked to indicate the extent of their agreement to various statements on a 4-point response scale ranging from strongly disagree (1) to strongly agree (4). All items were recoded in the same direction and responses were summed to provide five subscale scores. Cronbach’s alphas for the FWQQ subscales were 0.88 (demands), 0.79 (autonomy), 0.87 (equity), 0.75 (social resources), and 0.82 (caregiving rewards).

Work–family conflict was assessed with a 6-item measure, asking respondents to indicate the extent of agreement (from strongly disagree = 1 to strongly agree = 5) to each item [36]. Two subscale scores were obtained, with three items used to assess the degree that work interfered with family (e.g., “I have to miss family activities due to the amount of time I must spend on work responsibilities) and three items to assess the degree that family interfered with work (e.g., I have to miss work activities due to the amount of time I must spend on family responsibilities). Cronbach’s alphas were 0.59 and 0.54 for work-to-family conflict and family-to-work conflict, respectively.

Covariates

Covariates considered included mothers’ age (continuous), number of children (1, 2, 3, or more), the presence of at least one child aged five years or younger in the household (yes, no), and weekly paid work hours (31 h or less, 32–40 h, and 41+ h). Regarding the latter, all women who indicated that they were currently employed were eligible for the study. Small numbers of participants in some paid work hour categories, particularly those on the low end of weekly work hours, necessitated rather broad groupings for this variable.

### 2.3. Analyses

Frequencies and proportions (or mean/standard deviations) of study variables were determined, followed by bivariate analyses. Pearson correlation coefficients were calculated between potential mediators and psychological distress, followed by a univariate analysis of variance (general linear model) to test for mean differences in the study variables according to household income, adjusting for confounders.

Mediation analyses were performed using the PROCESS macro in SPSS v. 23 [37] (IBM Corp, Armonk, NY, USA). A series of simple mediation analyses were initially conducted, examining one potential mediator at a time, adjusting for confounders. As the primary independent variable, household income, was a categorical variable with four levels, three dummy coded variables were created, with the highest income group as the reference category. Non-parametric bootstrapping procedures using 5000 resamples were used to test the statistical significance of indirect effects of the proposed mediating variables. From these bootstrapped samples, point estimates and confidence intervals of indirect effects (total and specific) were estimated and calculated. Mediation was demonstrated if zero was not included within the 95% bias-corrected confidence interval, indicating that point estimates for indirect effects were statistically significant.

Variables that were statistically significant in the simple mediation analyses were then simultaneously entered into a multiple mediation analysis. In multiple mediation, specific indirect effects are calculated for each individual mediator, adjusting for other mediators in the model and confounders. Partially standardized indirect effects were also calculated to provide a measure of mediation effect size.

## 3. Results

The distribution for all study variables is shown in Table 1. Participants’ average age was 40 years, with the majority (63%) having two or more children in the household, and just over one-third reporting the presence of a young child (≤5 years). Nearly three-quarters of the sample spent 32 or more hours a week in paid work; 18% of the participants reported an annual household income of less than CAD CAD60,000. Table 2 shows low to moderate Pearson correlation coefficients in expected directions among potential mediators and psychological distress, with the strongest (0.53) between the two work–family conflict variables.

The patterning of study variables according to household income is shown in Table 3. An income–distress gradient was observed, with levels of psychological distress decreasing with increasing income. Among potential mediators, four variables were significantly associated with lower income: greater material deprivation, less equity in responsibility for domestic labor, lower job control, and greater work-to-family conflict.

Results from the simple mediation analysis identified the same four variables as mediators in the association between household income and psychological distress (Table A2).

Results of the multiple mediation analyses, with material deprivation, equity, job control, and work-to-family conflict simultaneously entered into the model, showed that compared to women in the highest income category (CAD CAD120,000+), those in the lowest (≤ CAD59,999) and 2nd lowest ( CAD60,000– CAD89,999) income categories reported significantly higher levels of psychological distress. Differences in psychological distress between the two upper income groups were not statistically significant; given that there was no difference in distress to explain, the remaining results that are presented below focus on women in the two lowest income groups relative to the highest.

**Figure 1:**
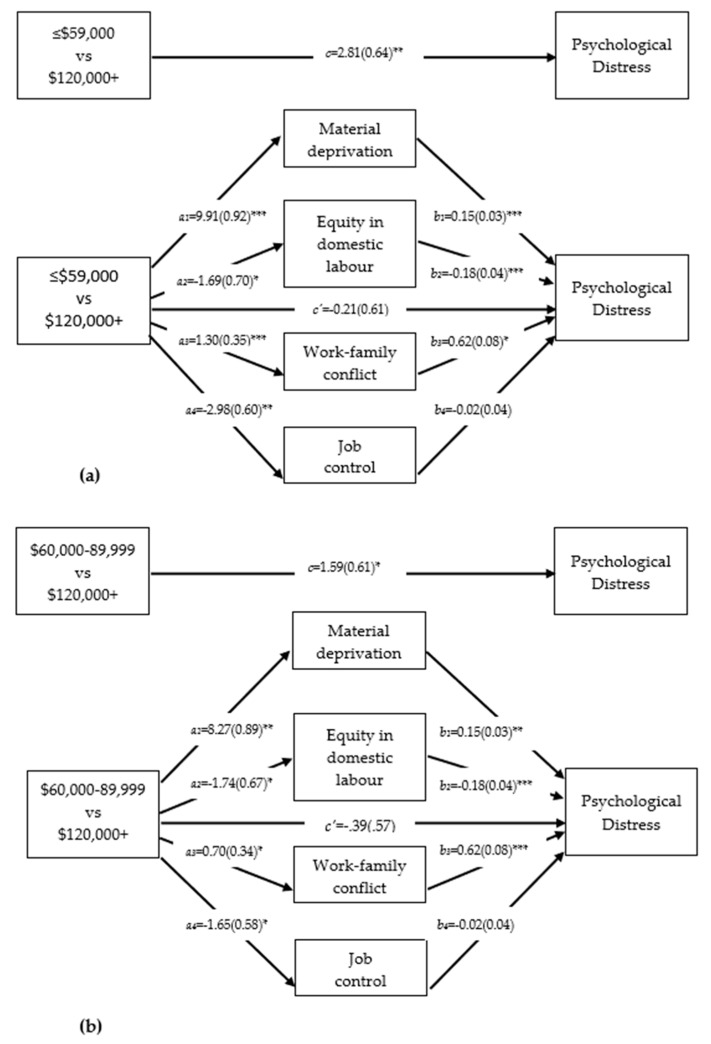
Results of multiple mediation analysis examining effects of household income on distress through material deprivation, equity, work–family conflict, and job control (*n* = 512).^1^ (**a**) Contrast between women in the ≤CAD 59,000 income group and those in the CAD120,000+ category; (**b**) Contrast between women in the CAD 60,000– CAD89,900 income group and those in the CAD 120,000+ category. ^1^ Adjusted for mothers’ age and paid work hours; * *p* < 0.05, ** *p* < 0.01, *** *p* < 0.001.

Figure 1 shows the results of the multiple mediation analyses for women in the highest income group compared to those in the ≤ CAD59,000 group (Figure 1a) and the CAD60,000– CAD89,999 group (Figure 1b). In both sets of results, women in the two lower income groups as compared to the higher income group reported: (1) greater psychological distress (path c); and (2) greater material deprivation, less equity in responsibility for domestic labor, greater work–family conflict, and lower job control (path a). With the exception of job control, all mediators were significantly related to psychological distress; that is, greater material deprivation, lower equity, and greater work–family conflict were each associated with higher psychological distress (path b). Table 4 further shows that for both of the lower income groups, the relative indirect effects of material deprivation, equity, and work–family conflict were statistically significant, whereas job control was not. That is, relative to women in the highest income group, those in groups 1 and 2 reported greater material deprivation, lower equity, and greater work–family conflict, each of which in turn were associated with greater psychological distress. As indicated by the partially standardized relative indirect values (Table 4), the largest mediation effect size was for material deprivation, followed by work–family conflict, and then equity in family work. Once adjusted for the mediators, the direct effect of income on distress was no longer statistically significant (Figure 1, path c’).

## 4. Discussion

In this study of employed partnered mothers, lower household income was associated with greater psychological distress, with material deprivation, work–family conflict, and inequity in responsibility for domestic work acting as mediators.

The observed association between decreased income and increased psychological distress in this study is consistent with previous research, both in general population [38,39] and employed samples of women [40,41]. It is important to note, however, that the findings of research examining the link between SES and mental health has not been invariant [42], and even in this study, there was no difference in psychological distress observed between women in the two upper income groups. However, a preponderance of evidence, including longitudinal, points to SES as an important determinant of mental health, with household income identified as one of the most robust predictors of all SES indicators, particularly for women [41,43].

Consistent with the results of the present study, the importance of material factors in explaining an SES gradient in mental health morbidity has been identified in other research with employed populations [44,45,46]. Some discussion in the literature has focused on whether material or psychosocial mechanisms best characterize this relationship [3]. Regarding material mechanisms, household income as an SES indicator is most strongly tied to the ability to buy health enhancing goods and services. Given that mental rather than physical health was the focus of this study, psychosocial mechanisms may be more critical in linking household income to women’s distress [3]. According to this perspective, being unable to meet (neo)material needs may be inherently stressful and/or lead to elevated distress through a process of social comparison [47].

Work–family conflict also contributed to the higher levels of psychological distress among the lower rather than higher income women in this study. Some previous research is in line with our finding, also documenting greater challenges among lower SES women in balancing the dual demands of work and life, and as one pathway that links lower social position to poorer mental health [9,48,49]. One explanation may be that workers with lower incomes may have less access to organizational practices and policies aimed at enhancing the negotiation of work and family responsibilities [50]. However, and in contrast to the results of this study, a more common finding is that work–life conflict may actually be more prevalent among higher as compared to lower status workers [12,51,52], and minimize rather than exacerbate SES inequities in mental health [13,14]. Schieman [12] used the phrase “stress of higher status” to describe higher SES individuals whose heavy work responsibilities may lead to a greater blurring of work–life boundaries, and in turn, the potential for conflict. These contrasting findings are difficult to reconcile, and more research is clearly needed. Work–life conflict is a relatively new addition to health inequities research, and research on the topic has been criticized for an overemphasis on the experiences of professional workers, including in the development of measures [53,54].

Women with lower income in this study reported less equity in responsibility for domestic labor, which in turn contributed to their higher levels of psychological distress. Perceived unfairness in paid work, conceptualized and measured in a variety of ways, has been linked to compromised mental health [55]. Though studied much less frequently, perceived unfairness in the division of family labor has also been associated with poorer mental health in women [20,21,22]. Equity theory posits that an imbalance between the contributions made and the benefits received in a close relationship can result in distress, particularly for the partner that is under-benefited [56]. Despite evidence of some gender convergence in domestic labor activities, women in heterosexual relationships, even when employed, continue to spend more time than men in unpaid family work [57,58]. It is important to keep in mind, however, that there is a difference between the division of family labor and the appraisal of that division, the latter of which was measured in this study. On the one hand, research indicates that the more time women spend completing unpaid tasks in the home relative to their spouses, the more unfair they consider the division of household labor to be [59]. On the other hand, the relationship is imperfect, as evidence suggests that many women who do more than their partner perceive such an arrangement as fair [59,60,61]. Thus, while the actual division of labor is an important determinant of perceived fairness, other microlevel and macrolevel factors are at play [60,61].

However, less research has examined perceptions of unfairness in family work in relation to household income; that is, why women with lower household income may perceive less equity in responsibility for domestic labor than higher income counterparts. Some research suggests that the number of daily hours women spend on housework decreases as household income increases [62,63]. Higher income women may have the financial resources to hire others to do the work and/or purchase more efficient household technology, thus reducing their own housework time and perceptions of overload [64]. However, if the above mechanism was in operation in the present study, we should have observed other measures of housework quality (i.e., demands and social resources) to also be patterned by household income (which were not). Likewise, as discussed above, there is a difference between the division of labor and how that division is perceived; to our knowledge, previous research has not examined how household income may impact the latter. Additional research is clearly needed to better understand the role of domestic labor in the genesis of SES inequities in women’s mental health.

### Strengths and Limitations

There were a number of strengths to this study. Using theoretically informed and psychometrically sound measures of exposure, we were able to examine a diverse array of material and psychosocial factors as potential mediators in SES differences in mental health. In addition to more mainstream mediators, the present study examined several novel exposures, including those related to domestic labor, a focus mostly absent in research aimed at elucidating mechanisms underlying SES–health disparities. Our consideration of work–family conflict and domestic labor in relation to mental health is also timely. COVID-19 has magnified mothers’ primary role in domestic labor within the family and the pandemic’s impact on their mental health, particularly when combined with paid work [65,66]; mothers experiencing material deprivation during the pandemic may be especially at risk of compromised mental health [65,66]. Although this study was conducted prior to the pandemic, our findings point to the importance of considering work–family conflict and domestic labor in future studies as determinants of mental health and as potential contributors to SES inequalities in women’s mental health.

Our findings also need to be considered in light of study limitations. The study design was cross-sectional; thus, the direction of association between exposures and outcome cannot be established. Due to common method bias, observed associations may be overestimated [67]. Our sample was recruited online which may result in a biased sample. However, research suggests that online surveys may produce estimates that are similar to those using more orthodox modes of data collection [68]. Furthermore, as the focus of this study was on estimating association rather than prevalence, representativeness is not of primary concern [69]. Although the vast majority of the study community had access to a computer at the time of the survey [70], the online requirement would have excluded the most socially and economically vulnerable women from participating. We did not collect information on other socio-demographic characteristics which may be associated with our study variables; for example, research suggests that the division of household labor is more equitable in Canadian-born couples compared to those born elsewhere [71], and among same sex couples compared to heterosexual [72]. Ethnicity is strongly associated with Canadian women’s SES and experience of material deprivation [73]. A larger sample combined with more detailed measurement would have permitted a more nuanced pattern of findings to emerge.

Finally, it is important to emphasize that although the data for this study was collected in 2012, we believe our findings remain relevant. Between 2012 and just prior to the pandemic, the gendered nature of paid and unpaid work roles in Canada changed little [57]. Evidently, COVID-19 and the resulting public health restrictions led to a rapid transformation in working life worldwide. For numerous parents, paid work, unpaid work, and child rearing/schooling at times co-occurred in the same domestic space. There was a modest increase in Canadian men’s participation in some household tasks during this time [74,75]; mothers, however, remained disproportionately responsible for unpaid household labor. Mothers of young children were identified as a particularly vulnerable group during the pandemic, with rates of anxiety and depression more than double compared to pre-pandemic times [76]. Consistent with the results of our study, material deprivation, low SES, work–family conflict, and the burdens of domestic labor continued to place women at an increased risk of poor mental health during the pandemic [65,66,77,78]. Knowledge of the mental health consequences of the pandemic continue to evolve and future research will confirm or refute whether the mediation model developed in this study will remain useful in understanding the poorer mental health of lower income working mothers compared to those more privileged.

## 5. Conclusions

In addition to material circumstances, work–family conflict and inequity in the division of household labor may partially explain how lower income translates into greater psychological distress for employed partnered mothers. To inform the development of policy aimed at enhancing women’s mental health, longitudinal research with diverse samples of women is required to elucidate how SES may differentially expose employed partnered mothers to hazards and resources within paid and domestic work contexts.

## Figures and Tables

**Table 1:** Characteristics of the study population (*n* = 512).

Variables	Number	% or Mean (SD)
Mothers’ age (yrs)	498	39.92 (7.21)
Number of children		
1 child	189	36.9
2 children	234	45.7
3 or more children	86	16.8
Child 5 years of age or younger in household		
No	335	65.4
Yes	177	34.6
Weekly paid work hours		
31 h or less	137	26.8
32–40 h	262	51.2
41 h or more	113	22.1
Household income (annual)		
≤ CAD59,999	92	18.0
CAD60,000– CAD89,999	102	20.5
CAD90,000– CAD119,999	130	26.2
CAD120,000+	119	23.4
Family work quality		
Demands	506	22.99 (4.32)
Autonomy	509	16.07 (2.86)
Equity	506	14.96 (4.90)
Social resources	497	8.55 (3.05)
Caregiving rewards	507	16.44 (2.79)
Paid work quality		
Demands	512	25.00 (4.29)
Decision latitude	512	26.82 (4.41)
Work–family interface		
Work-to-family conflict	512	7.82 (2.49)
Family-to-work conflict	512	7.10 (2.18)
Material deprivation	512	22.39 (7.34)
Psychological distress	512	12.39 (4.65)

**Table 2:** Correlations among key variables (*n* = 512).

Variables	1	2	3	4	5	6	7	8	9	10	11
1. Demands	1	−0.22 **	−0.43 **	−0.21 **	−0.19 **	0.36 **	−0.01	0.46 **	0.40 **	0.25 **	0.38 **
2. Autonomy		1	0.14 **	0.23 **	0.26 **	−0.04	0.11 *	−0.24 **	−0.29 **	−0.19 **	−0.29 **
3. Equity			1	0.22 **	0.36 **	−0.13 **	0.13 **	−0.26 **	−0.32 **	−0.26 **	−0.32 **
4. Social Resources				1	0.21 **	−0.11 *	0.04	−0.26 **	−0.14 **	−0.17 **	−0.28 **
5. Caregiving Rewards					1	0.03	0.15 **	−0.15 **	−0.25 **	−0.14 **	−0.25 **
6. Job Demands						1	0.18 **	0.38 **	0.14 **	0.17 **	0.20 **
7. Job Control							1	−0.13 **	−0.12 **	−0.24 **	−0.16 **
8. Work-to-Family Conflict								1	0.53 **	0.36 **	0.47 **
9. Family-to-Work Conflict									1	0.29 **	0.41 **
10. Material Deprivation										1	0.40 **
11. Psychological Distress											1

* *p* < 0.05, ** *p* < 0.01.

**Table 3:** The association of household income with psychological distress, material deprivation, and psychosocial qualities (means with standard errors in parentheses) (*n* = 512) ^1^.

Variables	≤CAD 59,999	CAD60,000– CAD89,999	CAD90,000– CAD119,999	CAD120,000+	*p* Value
Psychological distress	14.31 (0.47)	12.98 (0.45)	11.86 (0.40)	11.44 (0.42)	<0.001
Material deprivation	27.35 (0.68)	25.68 (0.64)	21.31 (0.57)	17.41 (0.60)	<0.001
Family work quality					
Demands	23.43 (0.45)	23.41 (0.43)	22.85 (0.38)	22.80 (0.40)	0.57
Autonomy	15.93 (0.31)	15.92 (0.29)	16.02 (0.26)	15.89 (0.27)	0.99
Equity	14.20 (0.51)	14.16 (0.48)	15.75 (0.43)	15.90 (0.46)	0.009
Social resources	8.61 (0.32)	8.55 (0.30)	8.59 (0.27)	8.28 (0.28)	0.84
Caregiving rewards	16.51 (0.29)	16.16 (0.28)	16.53 (0.24)	16.64 (0.26)	0.62
Job demands	25.40 (0.44)	25.42 (0.41)	24.68 (0.37)	25.10 (0.39)	0.50
Job control	25.25 (0.44)	26.59 (0.42)	27.14 (0.37)	28.30 (0.39)	<0.001
Work-to-family conflict	8.70 (0.26)	8.05 (0.24)	7.67 (0.22)	7.36 (0.23)	0.001
Family-to-work conflict	7.29 (0.23)	7.23 (0.22)	6.91 (0.19)	7.07 (0.20)	0.55

^1^ Adjusted for mothers’ age and paid work hours.

**Table 4:** Relative indirect effects and partially standardized indirect effects of income on psychological distress through material deprivation, equity in unpaid work, work–family conflict, and job control (*n* = 512) ^1^.

	Relative Indirect Effect	(95%CI)	Partially Standardized Relative Indirect Effect	(95%CI)
≤CAD 59,000 vs. CAD120,000+				
Material deprivation	1.45 (0.35)	0.79–2.18 *	0.31 (0.07)	0.17–0.47 *
Equity	0.30 (0.14)	0.06–0.61 *	0.07 (0.03)	0.01–0.13 *
Work–family conflict	0.81 (0.25)	0.35–1.33 *	0.18 (0.05)	0.08–0.29 *
Job control	0.04 (0.13)	−0.21–0.32	0.009 (0.03)	−0.05–0.07
CAD60,000–89,999 vs. CAD120,000+				
Material deprivation	1.21 (0.30)	0.64–1.85 *	0.26 (0.07)	0.14–0.40 *
Equity in domestic labor	0.31 (0.13)	0.08–0.59 *	0.07 (0.03)	0.02–0.13 *
Work–family conflict	0.43 (0.22)	0.04–0.88 *	0.09 (0.05)	0.01–0.19 *
Job control	0.04 (0.13)	−0.13–0.17	0.005 (0.02)	−0.03–0.04

^1^ Adjusted for mothers’ age and paid work hours; * *p* < 0.05.

## Data Availability

The data presented in this study are available on reasonable request from the corresponding author. The data are not publicly available due to ethical issues.

## Appendix A

**Table A1:** Family Work Quality Questionnaire (FWQQ).

The first set of questions that you will be asked on this survey are about what you think and feel about the family work that you do. We are defining family work as any “unpaid work done to maintain family members and/or a home” [79]. Family work includes that performed in and around the household including housework, yard work, gardening, and house maintenance. Family work also includes work related to “running the household” such as time spent planning meals, scheduling appointments for household members as well as the budgetary management of the home. Another important aspect of family work is that which focuses on the physical and/or emotional aspects of caregiving. Physical aspects include parenting of children (i.e., assisting with homework, driving children to activities, diapering, etc.), as well as elder care. Emotional aspects consist of work directed at meeting others’ emotional needs, improving their well-being, and maintaining harmony. Please indicate the degree to which you agree or disagree with various statements concerning your family work. Strongly disagree Somewhat disagree Somewhat agree Strongly agree When it comes to my family work… (item order randomized): Demands
I always feel pressured for time
It never seems to be done
It is a constant juggling act
I never seem to have enough time to complete what I need to do
I feel like I am being pulled in a million directions
There are a lot of conflicting demands on my time
I usually have to multitask
Autonomy
I decide when to do it
I control the speed at which I work
I establish plans for my own work
I am free to decide what work I will do
I decide on how much effort to put into it
Equity
The work is fairly divided up between me and my partner
I do too much of it compared to other family members
I wish my partner would contribute more
Responsibilities are not fairly divided in our household
I am satisfied with how family members share the work
I usually end up doing most of the work myself
I usually do most of the work that nobody else wants to do
Social resources
I can count on extended family to help if needed
I can count on friends to help if needed
I can rely if needed on support from within my community/neighborhood
I can count on people outside my household to provided unpaid assistance
Caregiving rewards For the last five statements, please focus specifically on your CAREGIVING responsibilities (rather than family work in general)
When it comes to my caregiving work… I enjoy the work
I find the work fulfilling
I find the work interesting
I feel good about it because I am helping the people I love
I feel appreciated by my loved ones for the work I do

**Table A2:** Simple mediation analyses of the effect of household income on psychological distress, mediated by variables measuring family work quality, job quality, the work–family interface, and material deprivation ^1^.

Mediators	a	b	Total Effect c	Direct Effect c’	Indirect Effect a x b (95%CI) ^2^
Demands					
≤CAD 59,999 vs. CAD 120,000+	0.65		2.96 ***	2.69 ***	0.27 (−0.43–0.52)
CAD 60,000–89,999 vs. CAD 120,000+	0.64		1.66 **	1.39 *	0.27 (−0.21–0.80)
CAD 90,000–119,999 vs. CAD 120,000+	0.08	0.42 ***	0.35	0.31	0.03 (−0.44–0.52)
Autonomy					
≤CAD 59,999 vs. CAD 120,000+	0.03		3.03 ***	3.04 ***	−0.01 (−0.45–0.40)
CAD 60,000–89,999 vs. CAD 120,000+	0.02		1.51 *	1.52 *	−0.01 (−0.40–0.39)
CAD 90,000–119,999 vs. CAD 120,000+	0.13	−0.51 ***	0.24	0.31	−0.07 (−0.44–0.30)
Equity					
≤CAD 59,999 vs. CAD 120,000+	−1.72 *		2.94 ***	2.39 ***	0.55 (9.11–1.03) *
CAD 60,000–89,999 vs. CAD 120,000+	−1.74 **		1.69 **	1.13	0.55 (0.15–0.99) *
CAD 90,000–119,999 vs. CAD 120,000+	−0.02	−0.32 ***	0.35	0.35	0.01 (−0.40–0.40)
Social resources					
≤CAD 59,999 vs. CAD 120,000+	0.30		3.02 ***	3.16 ***	−0.14 (−0.57–0.26)
CAD 60,000–89,999 vs. CAD 120,000+	0.25		1.71 **	1.83 **	−0.12 (−0.58–0.17)
CAD 90,000–119,999 vs. CAD 120,000+	0.40	−0.48 ***	0.37	0.56	−0.19 (−0.58–0.18)
Caregiving rewards					
≤CAD 59,999 vs. CAD 120,000+	−0.16		2.97 ***	2.90 ***	0.08 (−0.29–0.44)
CAD 60,000–89,999 vs. CAD 120,000+	−0.50		1.59 *	1.35 *	0.24 (−0.11–0.60)
CAD 90,000–119,999 vs. CAD 120,000+	−0.04	−0.48 **	0.28	0.26	0.02 (−0.33–0.33)
Job demands					
≤CAD 59,999 vs. CAD 120,000+	0.32		3.00 ***	2.91 ***	0.09 (−0.22–0.46)
CAD 60,000–89,999 vs. CAD 120,000+	0.35		1.63 **	1.53 *	0.09 (−0.21–0.42)
CAD 90,000–119,999 vs. CAD 120,000+	−0.39	0.27 ***	0.26	0.37	−0.11 (−0.40–0.16)
Job control					
≤CAD 59,999 vs. CAD 120,000+	−2.91 ***		3.00 ***	2.67 ***	0.33 (0.04–0.71) *
CAD 60,000–89,999 vs. CAD 120,000+	−1.59 **		1.63 *	1.45 *	0.18 (0.02–0.41) *
CAD 90,000–119,999 vs. CAD 120,000+	−1.21 *	−0.11 *	0.26	0.12	0.14 (0.004–0.34) *
Work-to-family conflict					
≤CAD 59,999 vs. CAD 120,000+	1.37 **		3.00 **	1.78 **	1.22 (0.56–1.92) *
CAD 60,000–89,999 vs. CAD 120,000+	0.71 *		1.63 *	1.00	0.63 (0.08–1.22) *
CAD 90,000–119,999 vs. CAD 120,000+	0.25	0.89 ***	0.26	0.04	0.22 (−0.34–0.78)
Family-to-work conflict					
≤CAD 59,999 vs. CAD 120,000+	0.22		3.00 ***	2.81 ***	0.19 (−0.32–0.70)
CAD 60,000–89,999 vs. CAD 120,000+	0.16		1.63 **	1.50 **	0.14 (−0.32–0.61)
CAD 90,000–119,999 vs. CAD 120,000+	−0.18	0.85 ***	0.26	0.42	−0.15 (−0.63–0.33)
Material deprivation					
≤CAD 59,999 vs. CAD 120,000+	9.92 ***		3.00 ***	0.34	2.66 (1.94–3.42) *
CAD 60,000–89,999 vs. CAD 120,000+	8.21 ***		1.63 *	−0.57	2.20 (1.54–2.93) *
CAD 90,000–119,999 vs. CAD 120,000+	3.70 ***	0.27 ***	0.26	−0.73	0.99 (0.55–1.48) *

^1^ All analyses adjusted for women’s age and paid work hours; ^2^ Bias corrected bootstrap results for the indirect effect, number of resamples is 5000. * *p* < 0.05, ** *p* < 0.01, *** *p* < 0.001.
